# Ultrasensitive Hydrogen Detection Using GNRFET Sensor: Multimetric Optimization via Geometry, Temperature, and Oxygen Environment

**DOI:** 10.3390/mi17050632

**Published:** 2026-05-21

**Authors:** Mohammad K. Anvarifard, Zeinab Ramezani

**Affiliations:** 1 Department of Engineering Sciences, Faculty of Technology and Engineering, East of Guilan, University of Guilan, Rudsar 4489163157, Iran; 2 Department of Electrical and Computer Engineering, College of Engineering, University of Miami, Miami, FL 33146, USA

**Keywords:** GNRFET gas sensor, multi-metric sensitivity, transfer characteristic, threshold voltage, structural and environment engineering

## Abstract

This work presents a comprehensive analysis of a Palladium (Pd)-gated graphene nanoribbon field-effect transistor (GNRFET) as a high-sensitivity potential hydrogen sensor under idealized conditions, focusing on the structural and environmental control of multimetric sensitivity. Hydrogen adsorption is modeled through pressure-dependent work-function modulation and interface coverage, including competition with oxygen. For hydrogen gas at a pressure of $P_{H_{2}}=10^{-6}$ Torr without O_2_, the sensor exhibits a maximum threshold voltage sensitivity of about 300 mV, which is reduced to roughly 40 mV under an oxygen partial pressure of 152 Torr, quantifying the impact of background gas on response. Band diagrams, transmission spectra, local density of states, and transfer characteristics are examined over wide ranges of H_2_ pressure, temperature, gate length, and nanoribbon width. Sensitivity is evaluated using drain current change, threshold voltage shift, and average subthreshold swing variation. Results showed that the sensitivity based on current is high for ultralow hydrogen pressures, whereas it is low in higher levels of pressure compared to the sensitivity based on subthreshold. Also, uncertainty analysis revealed that the threshold voltage metric remains largely geometry-independent and thus tolerant to fabrication variations.

## 1. Introduction

Gas sensors play a critical role in environmental monitoring, chemical process control, agriculture, and medical applications [1]. The increasing demand for environmental safety and healthcare has led to significant research into the development of highly sensitive and selective smart gas sensors [2]. These devices are essential for detecting hazardous gas leaks and monitoring ambient air quality, preventing accidents, and ensuring proper gas mixtures in various settings [3,4]. The evolution of semiconductor-based microsensors/microdevices into nanodevices has been driven by the necessity for measurements at the nanoscale [5,6].

There is significant interest in improving the performance of field-effect transistor (FET)-based sensors. FET gas sensors have attracted substantial interest due to their compact size, low energy consumption, and compatibility with CMOS technology [7]. Nanomaterials are excellent transducers for gas detection within FET sensors, improving performance in areas such as sensitivity, response time, selectivity, stability, and energy consumption, while also enabling operation at ambient temperature [8,9]. This technology is particularly suitable for miniaturized gas detection systems and is attracting increasing attention for sophisticated functionalization in response to external stimuli [10]. FETs are considered an extremely efficient platform for gas and volatile organic compound (VOC) sensing due to their miniaturized form factor, high sensitivity, and ultra-low power consumption [11]. They are crucial for enabling sophisticated modern lifestyles and ensuring safe working environments by detecting a wide range of analytes, including gaseous species, ionic compounds, and biological molecules [10].

Sensing H_2_ gas is necessary due to its explosive nature above 4% concentration, low ignition energy, and odorless properties, which pose severe safety risks in fuel cell vehicles and hydrogen infrastructure. Reliable sensors enable leak detection, prevent explosions, and ensure compliance with safety standards, as emphasized in comprehensive reviews [12,13].

Field-effect transistor (FET) gas sensors, particularly those based on bulk materials such as silicon, can be used as baseline devices for sensing since they offer high flexibility and compatibility with CMOS technology, enabling improvements in electrical performance [14,15,16,17]. Despite this main advantage, it faces several fundamental limitations that impact its performance and widespread application as a gas sensor.

One significant challenge for bulk material FET gas sensors is their inherently low surface-to-volume ratio. In contrast, one-dimensional (1D) nanostructures offer a large surface area-to-volume ratio, which is a key advantage for achieving high sensitivity and fast response in gas sensors [18]. This characteristic allows for more interaction sites with target gas molecules, enhancing the sensing capabilities. For instance, nanomaterials and nanostructures improve sensor sensitivity and response time due to their high surface-to-volume ratio. This property is crucial as it directly affects the number of active sites available for gas molecule adsorption [19,20,21].

High subthreshold swing (SS) is another limitation for bulk silicon FET gas sensors. A steep subthreshold slope is desirable for low-power operation and high sensitivity [22]. The subthreshold swing can be influenced by the gate oxide thickness and doping concentration. While advanced designs, such as tunnel FETs and ferroelectric FETs, aim to achieve a sub-60 mV/decade subthreshold swing, conventional bulk silicon devices often struggle with this characteristic. This high subthreshold swing can lead to an undesirable rise in the off-current [23,24].

Despite advances, FET gas sensors can exhibit limited sensitivity and detection limits for specific gases and concentrations [25]. The so-called “Boltzmann Tyranny” effect can impose limitations on achievable sensitivity [26]. While some carbon nanotube (CNT) sensors show linear responses for concentrations from sub-ppm to hundreds of ppm, achieving ultra-low detection limits consistently across various analytes remains a significant hurdle. Efforts to improve sensitivity often involve material optimization, such as using molybdenum disulfide (MoS_2_)-based FET sensors [19,27].

Advanced gas sensors based on nanomaterials offer important advantages for precise detection in safety-critical applications like hydrogen infrastructure and environmental monitoring. Graphene nanoribbon FETs (GNRFETs) on SiC substrates enable superior oxygen and gas sensitivity through enhanced carrier modulation and high surface-to-volume ratio, achieving room-temperature operation with low power consumption [28]. MoS_2_-based sensors provide exceptional H_2_ selectivity and rapid response via large active surfaces, noble metal decoration, and nanocomposites, essential for leak detection in clean energy systems [29]. Ni-doped In_2_O_3_ nanostructures exhibit ultrafast NO_2_ sensing at low temperatures due to optimized defect sites and electronic sensitization, overcoming bulk sensor limitations in selectivity and recovery time [30]. These innovations collectively deliver high sensitivity, stability, and energy efficiency, addressing challenges in traditional sensors for real-time industrial and health monitoring [30].

Although gas sensors based on nanomaterials can be introduced as an effective solution to supersede the limitations of using bulk materials, they still inherit several serious challenges, like those seen for bulk materials, marked as slow response and recovery time and high operating temperatures.

The FET gas sensors can suffer from slow response and recovery times, particularly for certain gases. For example, studies have shown that it can take approximately one hour for an FET-type gas sensor to fully recover at 180 °C [19]. This slow recovery is often attributed to the kinetics of gas adsorption and desorption on the sensing material, as well as the chemical reactions involved. While various methods, like pulse biasing schemes, have been explored to improve recovery, it remains a considerable challenge [31,32].

Many FET gas sensors, especially those utilizing metal oxides, require high operating temperatures for optimal performance. Operating temperatures can reach up to 775 °C for some catalytic metal–insulator silicon carbide FETs [33]. These high temperatures are often necessary to facilitate surface reactions with gases, achieve quicker equilibration times, and ensure stable operation [34]. However, high operating temperatures lead to increased power consumption, reduced device longevity, and limit their integration into low-power, portable devices [35]. Some research indicates that while room-temperature gas sensors have seen recent advancements, the operating temperature of gate-sensitive FET gas sensors often remains high [35].

The detection and monitoring of hydrogen (H_2_) gas have garnered significant attention due to hydrogen’s growing role as a clean energy carrier and its inherent safety risks associated with flammability and explosiveness. Precise and reliable H_2_ gas sensors are critical for ensuring safe operation across numerous applications, including fuel cells, industrial processes, and hydrogen infrastructure [36]. Despite advances in sensor technologies, challenges remain in optimizing sensor performance metrics such as sensitivity, selectivity, response time, and operational stability under varying environmental and device parameters [37].

Recent studies demonstrate that the performance enhancement of graphene-based gas sensors is primarily achieved through surface engineering and chemical modification. Palladium nanoparticle decoration on graphene via electrodeposition significantly improved the ammonia sensing performance, with DFT calculations attributing the enhancement to increased charge transfer and optimized adsorption sites [37]. Another study employed first-principle calculations and experiments to show that co-doping graphene with palladium and epoxy groups leads to stronger SO_2_ adsorption and superior sensing response compared to singly modified structures [38]. In a further investigation, palladium-cluster-decorated graphene was examined for inert gas detection, where weak adsorption-induced charge transfer effects governed the sensing mechanism and enabled selective responses depending on cluster size [39]. Overall, these works highlight that sensor performance is strongly governed by surface functionalization strategy, metal cluster size, and adsorption–charge transfer interactions.

The aim of this study is to explore the fundamental device physics limits under idealized conditions. The ballistic regime allows isolation of intrinsic sensing mechanisms. This study systematically investigates the sensitivity dependence of H_2_ gas sensors based on graphene nanoribbon field-effect transistors (GNRFETs) on key factors, including operating voltage, temperature, hydrogen gas pressure, and the presence or absence of oxygen gas at different partial pressures, by approaching practical conditions. Additionally, device-specific parameters, including the gate length and GNRFET channel width, are explored to evaluate their impact on sensor response. Sensitivity metrics are rigorously defined via quantifiable electrical characteristics such as average subthreshold swing, drain current modulation, and threshold voltage shifts, offering comprehensive insights into transduction mechanisms under varying conditions [40].

The inclusion of oxygen gas at differing pressures enables a realistic evaluation environment, mimicking ambient conditions and surface chemistry effects that critically influence adsorption and sensor dynamics. Such extensive parameterization is vital, as demonstrated in this study, because it addresses sensor behavior not only in idealized conditions but also in complex, multicomponent gas mixtures likely encountered in practical applications [41].

Previous studies on FET-based hydrogen sensors, particularly graphene and GNR-based devices, capture general sensing trends [26,36,40,41,42]. They often do not provide a comprehensive comparison across different sensitivity metrics, nor do they account for the impact of background gases. In this work, we address these limitations by introducing a multimetric sensitivity framework based on threshold voltage shift, drain current modulation, and subthreshold swing variation. In addition, hydrogen sensing is modeled in the presence of oxygen through pressure-dependent interface coverage, enabling a more realistic evaluation of sensor response. Furthermore, an uncertainty-aware analysis is incorporated to assess the robustness and reliability of different sensing metrics under parameter variations. This combined approach provides deeper physical insight and more practical guidance for sensor design compared to prior deterministic studies.

By comprehensively evaluating these factors, this work contributes valuable insights toward the design and optimization of high-performance H_2_ gas sensors. The findings are expected to enhance sensor accuracy, reliability, and safety margins in hydrogen detection systems, ultimately advancing the deployment of hydrogen technologies with greater confidence in monitoring capabilities. This study underscores the fundamental and practical significance of analyzing parameter-dependent sensor behavior to drive innovations in nanoelectronic gas-sensing platforms.

## 2. GNRFET Gas Sensor and Sensing Mechanism

**Figure 1:**
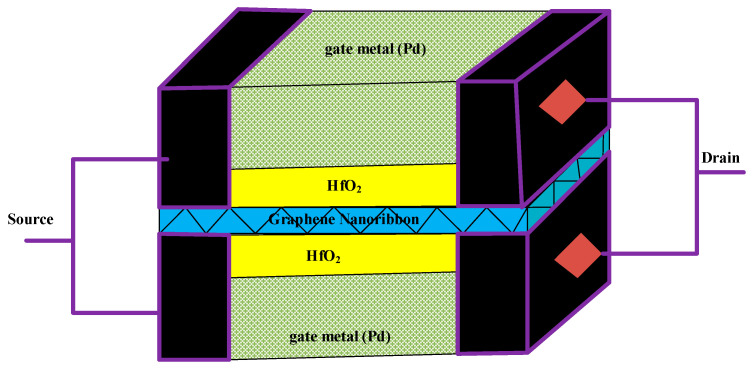
A schematic of the GNRFET gas sensor under study.

Figure 1 shows a three-dimensional (3D) view of the GNRFET device implemented as a hydrogen gas sensor. A graphene nanoribbon with an index of 12 (NT = 12), corresponding to a bandgap energy of 0.735 eV, is used as the channel material. The physical gate length is tuned to an ultrascaled value of 16 nm, enabling ballistic transport within a quantum-mechanical framework. To increase the electrostatic coupling between the gate and channel region, hafnium oxide (HfO_2_) with a relative permittivity of 16 is deposited on the GNR layer with a thickness of 1.5 nm. The GNRFET gas sensor under study features an intrinsic channel region and heavily doped source/drain regions at a level of 2.86 × 10^8^ m^−1^ and utilizes Pd metal with a work function of 4.8 eV as the sensing element to capture hydrogen atoms at the metal/oxide interface. The operating conditions for the gas sensor are an ambient temperature of 250 K, a drain bias of *V_D_* = 0.5 V, and a gate voltage swept from 0 to 1 V. These conditions are assumed for the evaluation of the gas sensor unless otherwise stated.

**Figure 2:**
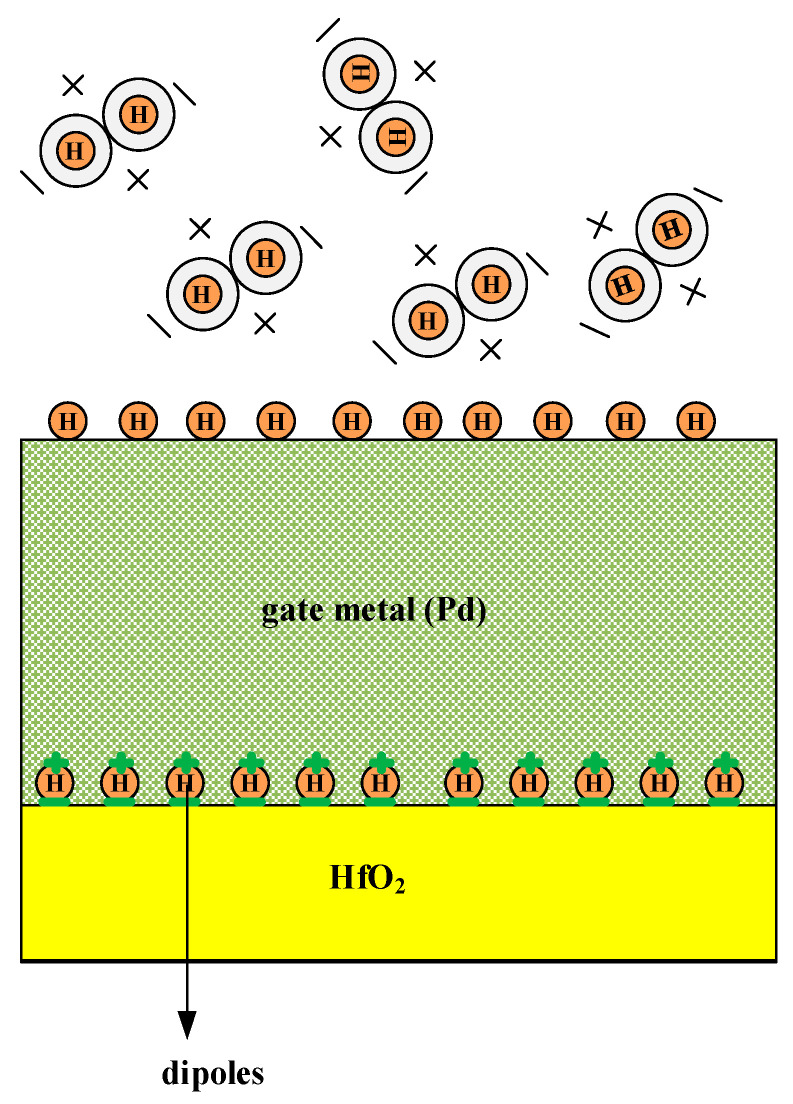
A demonstration of catalytic dissociation of H_2_ molecules at a metal surface. A dipole layer is formed at the interface of the gate oxide and the metal, resulting in a change in the Pd metal work function.

To gain deeper insight into how the GNRFET gas sensor detects H_2_ gas, Figure 2 illustrates the physical mechanism of hydrogen-atom adsorption at the metal/oxide interface. In the GNRFET gas sensor, hydrogen detection relies on the catalytic dissociation of H_2_ molecules at the metal surface (Pd metal) [26]. The resulting atomic hydrogen diffuses to the metal–insulator interface, forming an electric dipole layer. This interfacial dipole directly modulates the gate’s work function (*W_F_*), which transduces the chemical signal into a measurable shift in the transfer characteristics of the GNRFET device.

The adsorption and diffusion processes are governed by gas kinetics and thermodynamics. The impinging gas flux, W, is determined by pressure (P), molecular mass ($m_{g}$), and temperature (T): $W=P/\sqrt{2\pi m_{g}k_{B}T}$. The thermodynamic driving force is described by the heat of adsorption at the interface, $\Delta H_{i}$, which itself depends on the interfacial hydrogen coverage, $n_{i}$, following a Temkin isotherm model: $\Delta H_{i}=\Delta H_{i0}(1-\alpha n_{i})$. Here, α is a coupling constant, $\Delta H_{i0}$ is the initial heat of adsorption, and $n_{i}$ represents the fractional coverage of available adsorption sites with concentration *N_i_*.

The resultant change in the gate work function, which is the core sensing metric, is quantitatively expressed as follows [26]:

(1) $$\Delta W_{F}=-{\left(\varepsilon \right)}^{-1}n_{i}N_{i}lq$$

where l is the effective dipole length, q is the elementary charge, and ε is the permittivity. This relationship directly links the interfacial hydrogen coverage to the electronic parameter governing the GNRFET sensor operation. Since the gate is metallic, the surface and interface coverage are reduced due to the presence of oxygen gas in the ambient. As a result, $\Delta W_{F}$ is reduced owing to water formation and subsequent reactions.

The multimetric sensitivity analysis is performed on the gas sensor platform, thus delivering vital information on sensing behavior. Three metrics in terms of threshold voltage sensitivity *S_VTH_*, drain current sensitivity percentage *S_ID_*, and average subthreshold swing sensitivity percentage *S_Sub_* have been introduced as the multimetric measurements of the GNRFET gas sensor. Specifically, the gas sensor is monitored before and after gas injection, and the transfer characteristics are measured. These multimetric parameters are defined as follows:

(2) $$S_{VTH}=\left|V_{TH}^{No-Gas}-V_{TH}^{Gas}\right|$$

(3) $$S_{ID}=\left(\frac{I_{D}^{No-Gas}-I_{D}^{Gas}}{I_{D}^{No-Gas}}\right)*100\;\%$$

(4) $$S_{Sub}=\left(\frac{Swing^{No-Gas}-Swing^{Gas}}{Swing^{No-Gas}}\right)*100\;\%$$

In all the relations above, subscripts *No-Gas* and *Gas* denote the sensor operating before and after exposure to H_2_ gas, respectively. Threshold voltage (*V_TH_*) in Equation (2) is the gate voltage corresponding to the drain current of 1 × 10^−6^ A/µm. *I_D_* in Equation (3) is the ON drain current at the *V_G_* = 1 V and *V_D_* = 0.5 V. Regarding Equation (4), the variable Swing is the average subthreshold swing at the range of zero gate voltage and threshold voltage.

## 3. Computational Method

The operational characteristics of the graphene nanoribbon field-effect transistor (GNRFET) were modeled through a self-consistent numerical scheme that couples electrostatics and quantum transport. This computational framework simultaneously resolves the two-dimensional Poisson equation for electrostatic potential distribution and employs the non-equilibrium Green’s function (NEGF) formalism, grounded in the Schrödinger equation, to describe ballistic carrier injection and propagation. This dual approach accurately captures gate-modulated channel potential while treating source-to-drain transport quantum mechanically.

The electrostatic landscape is governed by the Poisson equation in two dimensions:

(5) $$\nabla .\left(\nabla \varepsilon \varphi \right)=-q\rho$$

Here, φ denotes the electrostatic potential, ϵ is the permittivity, and ρ the net charge density per unit area. The charge term is expressed as:

(6) $$\rho =p-n+N_{D}-N_{A}$$

where q is the elementary charge, p and n are hole and electron densities, and $N_{D}$ and $N_{A}$ are ionized dopant concentrations.

The Hamiltonian (H) for the GNR channel was constructed using an atomistic, single-orbital tight-binding model considering nearest-neighbor interactions, with a hopping integral $t_{0}=2.7$ eV. To achieve computational efficiency, the real-space Hamiltonian was transformed into a decoupled mode-space representation, significantly reducing matrix dimensions while preserving accuracy.

The retarded Green’s function matrix, which encodes the system’s electronic structure under non-equilibrium conditions, is calculated as:

(7) $$G^{r}(E)={\left[\left(E+i0^{+}\right)-H-U-\sum_{S}^{r}E-\sum_{D}^{r}E\right]}^{-1}$$

here E is energy, I is the identity matrix, U is the potential energy matrix from the Poisson solution, and $\Sigma_{S(D)}^{r}$ are the contact self-energy matrices incorporating boundary conditions for the source and drain reservoirs. A small imaginary part $i0^{+}$ ($10^{-5}$ eV) ensures numerical stability.

The coupling broadening matrices are given by $\Gamma_{S(D)}=i(\Sigma_{S(D)}^{r}-\Sigma_{S(D)}^{r†})$. These define the energy-dependent electron transmission probability:

(8) $$T(E)=\mathrm{Trace}[\Gamma_{S}(E)G^{r}(E)\Gamma_{D}(E)G^{r†}(E)]$$

The quantum-mechanical electron density is computed by integrating the spectral function contributions from both contacts:

(9) $$n(x)=\frac{1}{2\pi }\int_{-\infty }^{\infty }\left[G^{r}\Gamma_{S}G^{r†}f(E-\mu_{S})+G^{r}\Gamma_{D}G^{r†}f(E-\mu_{D})\right]dE$$

where $f(E-\mu_{S(D)})$ is the Fermi–Dirac distribution function at the source (drain) Fermi level $\mu_{S(D)}$. This charge density, n(x), is fed back into the Poisson equation. The coupled Poisson–NEGF system is solved iteratively until convergence is achieved in both the potential profile φ and the charge density n.

Upon convergence, the steady-state drain current is evaluated via the Landauer formula:

(10) $$I_{D}=\frac{2e}{h}\int_{-\infty }^{\infty }T(E)[f(E-\mu_{S})-f(E-\mu_{D})]dE$$

This formalism ensures a fully quantum-mechanical, self-consistent description of electrostatics and ballistic transport in the nanoscale device.

This study employs a self-consistent Poisson–NEGF framework under the assumption of ballistic transport to describe the intrinsic behavior of short-channel GNRFETs. While this approach captures the fundamental transport physics, it does not include scattering processes such as phonon interactions, impurity effects, or structural disorder, which can influence current levels and subthreshold characteristics in realistic devices. In addition, trap states and interface-related charge effects are not explicitly considered and may impact threshold voltage stability and noise behavior. Hydrogen adsorption and oxygen competition are represented through an effective pressure-dependent surface coverage model coupled with work-function modulation. Although this approach reflects the dominant sensing mechanism, it does not resolve detailed surface reaction pathways. Moreover, contact resistance and Schottky barrier effects are not explicitly included. Therefore, the results should be interpreted as indicative of intrinsic device trends rather than exact predictions for experimental implementations.

## 4. Evaluation of Sensing Performance

**Figure 3:**
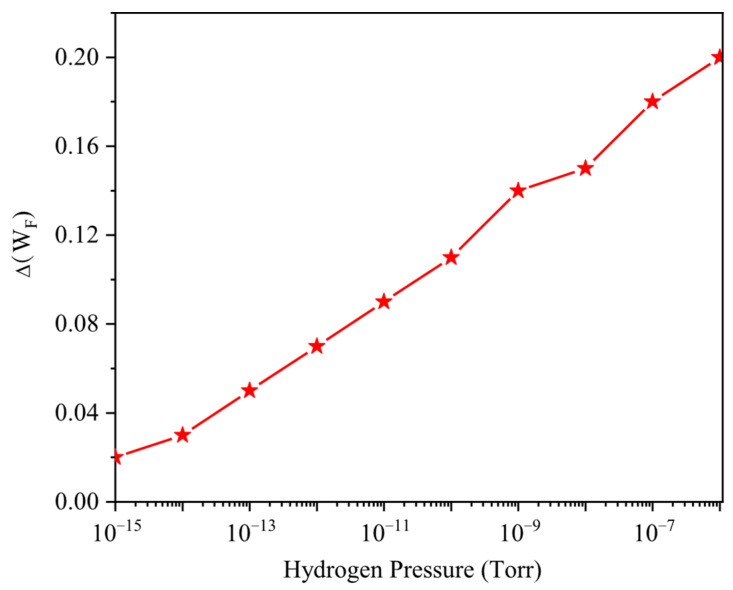
Work-function shift Δ*W_F_* as a function of hydrogen pressure ranging from 10^−15^ to 10^−6^ Torr. The temperature is 250 K.

The formation of dipoles at the interface of the oxide and gate metal is the main reason why the transport is changed. As explained in Section 2, the gate metal work function is shifted since the dipoles at the interface modify the Fermi level [42]. Figure 3 illustrates the exponential increase in the Pd gate work-function shift $\Delta W_{F}$, with rising hydrogen pressure from $10^{-15}$ to $10^{-7}$ Torr under oxygen-free conditions at 250 K. The figure demonstrates the strong sensitivity of the Pd surface to ultralow H_2_ concentrations, enabling a significant modulation of the GNRFET electrostatics even at sub-trace hydrogen levels. As expected from Equation (1), the work-function shift correlates directly with the hydrogen concentration, as illustrated in the figure.

**Figure 4:**
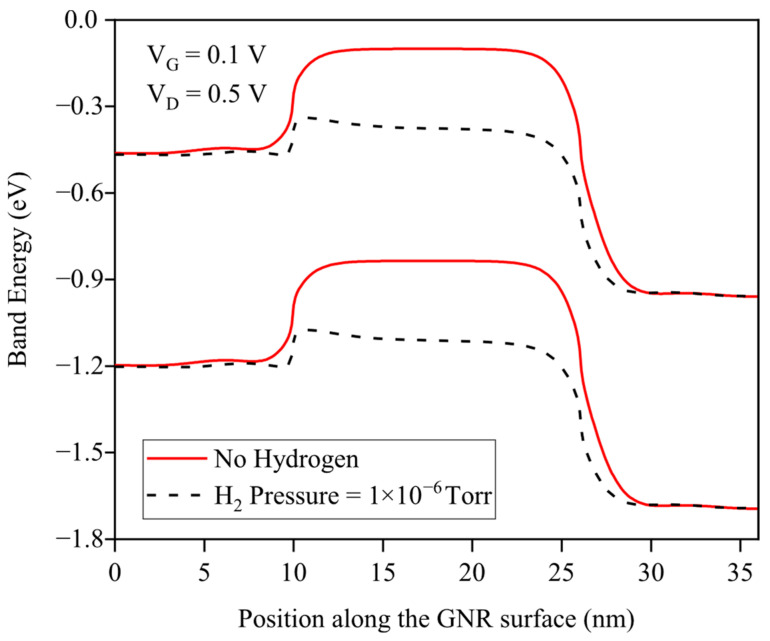
Conduction and valence band energy profile along the GNR surface before and after an injection of H_2_ gas.

Figure 4 demonstrates the band energy diagram along the GNR channel at $V_{G}=0.1\;V$ and $V_{D}=0.5\;V$, revealing that hydrogen adsorption substantially reduces the potential barrier height, facilitating enhanced carrier transmission across the channel and thereby increasing drain current—a key mechanism enabling the GNRFET’s high hydrogen sensitivity. Hydrogen chemisorption on the Pd gate follows Sieverts’ law, changing the work function exponentially with pressure as illustrated in Figure 3. This electrostatically modulates the GNR Fermi level, lowering the source–channel potential barrier by ≈0.3 eV as seen in Figure 4, which enhances carrier transmission exponentially. Operating at 250 K in the subthreshold regime maximizes sensitivity, as small work-function shifts cause exponential drain current changes. The tight Pd-GNR electrostatic coupling and hydrogen diffusion at reduced temperature enable ppb-level detection limits and response stability.

**Figure 5:**
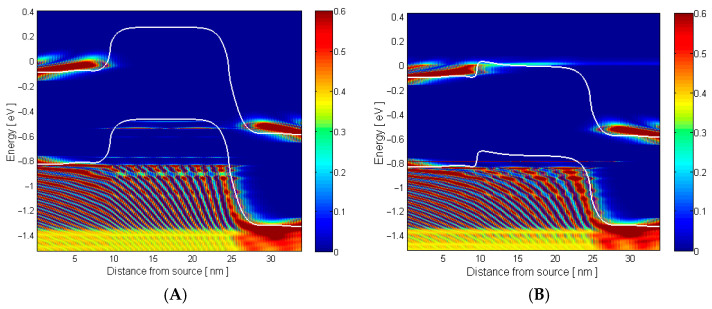
LDOS dependence on the energy position of the GNRFET gas sensor for (**A**) before injection and (**B**) after injection of H_2_ gas.

To better understand the fundamental and key mechanism that is responsible for sensing H_2_ gas, Figure 5 presents the energy-resolved local density of states (LDOS) along the GNR channel before and after hydrogen gas injection, where the color intensity depicts carrier concentration. Notably, hydrogen exposure substantially increases transmission probability in the subthreshold region near the source, evidenced by the enhanced red/yellow coloration, enabling more carriers to traverse the channel and contributing to the observed drain current amplification in the GNRFET hydrogen sensor. The intensified LDOS reflects reduced backscattering and improved coupling to channel modes. Charge accumulation at the source sharpens potential gradients, especially at 250 K, where thermal broadening is minimal. The exponential transmission sensitivity to small barrier reductions (approximately 0.3 eV from hydrogen adsorption) explains the potential ppb-level detection limits without the need for exotic materials; the one-dimensional GNR geometry concentrates this modulation into measurable current amplification.

**Figure 6:**
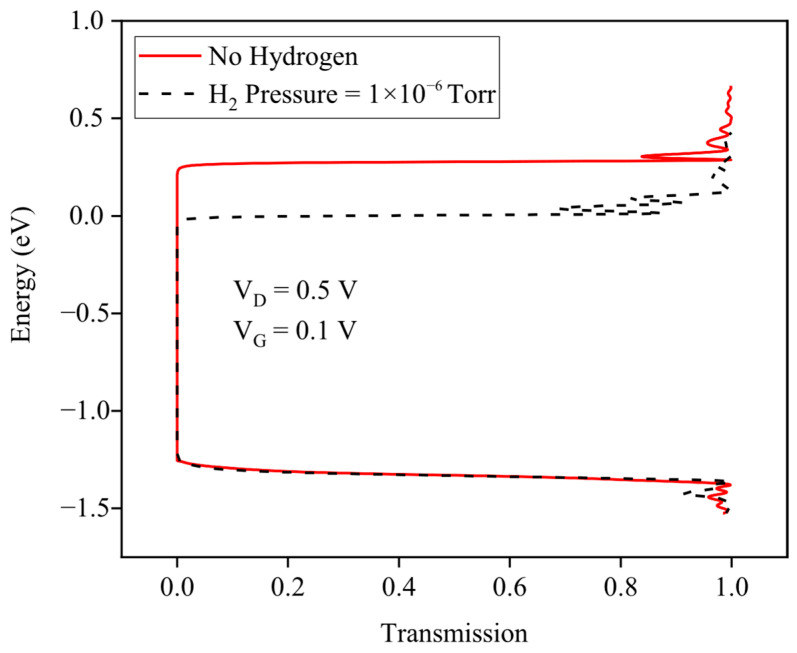
The energy-transmission profile at H_2_ pressure of 1 × 10^−6^ Torr.

To further complete the explanation, Figure 6 reveals that hydrogen injection dramatically elevates subthreshold transmission across the entire energy range (−1.5 to 0 eV), with an emergence of sharp resonant peaks representing quasi-bound state activation in the flattened channel potential. The baseline transmission increase at low energies is critical. It shifts more states into the narrow Fermi window at 250 K, enabling exponential current amplification. Multisubband resonant activation, unique to one-dimensional GNR confinement, converts the modest 0.3 eV barrier reduction into superlinear transport changes. Preserved quantum interference features confirm ballistic operation, ensuring ppb sensitivity through pure electrostatic modulation without scattering degradation.

**Figure 7:**
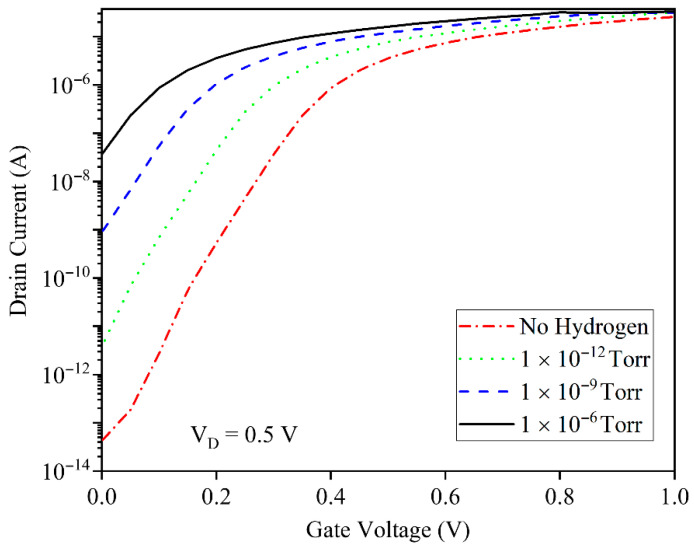
The drain current as a function of the gate voltage before and after the gas injection. The different hydrogen pressures have been assumed.

Figure 7 demonstrates the transfer characteristics ($I_{D}$ vs. $V_{G}$) at $V_{D}=0.5$ V across hydrogen pressures from baseline to $1\times 10^{-6}$ Torr. The subthreshold region exhibits dramatic current enhancement with increasing H_2_ exposure, indicating exponential sensitivity to hydrogen-induced barrier lowering. Notably, the subthreshold swing sharpens progressively with hydrogen injection, while ON-state current modulation remains modest, indicating that GNRFET operates optimally as a subthreshold-regime hydrogen sensor with minimal saturation current degradation.

**Figure 8:**
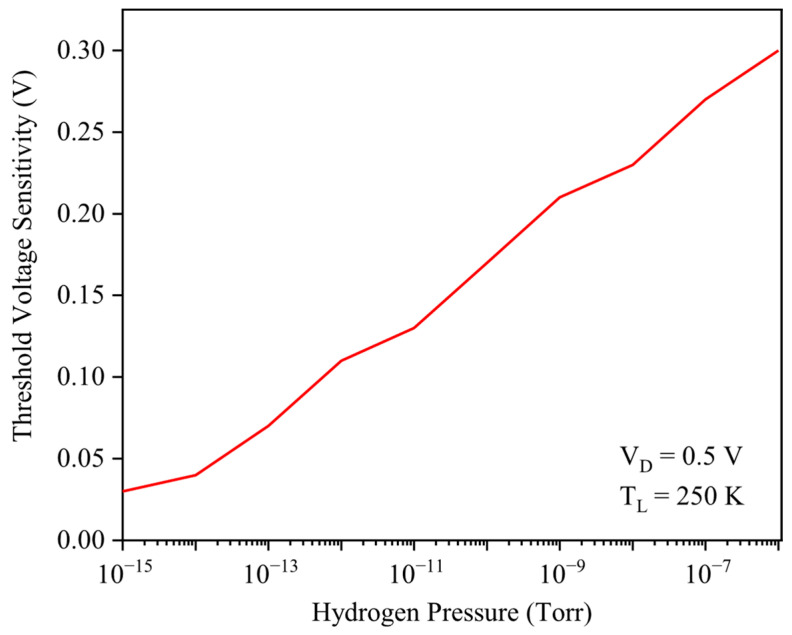
Threshold voltage sensitivity to the hydrogen pressure.

The threshold voltage sensitivity of the GNRFET as a function of hydrogen pressure from $10^{-15}$ to $10^{-6}$ Torr has been demonstrated in Figure 8. It exhibits a nearly constant positive slope that indicates an almost linear sensor response versus pressure. This $\Delta V_{TH}$ is linear and steadily increasing with pressure, which demonstrates that the device maintains high sensitivity even at ultra-low H_2_ levels, confirming its suitability for quantitative, low-concentration hydrogen detection in practical applications.

**Figure 9:**
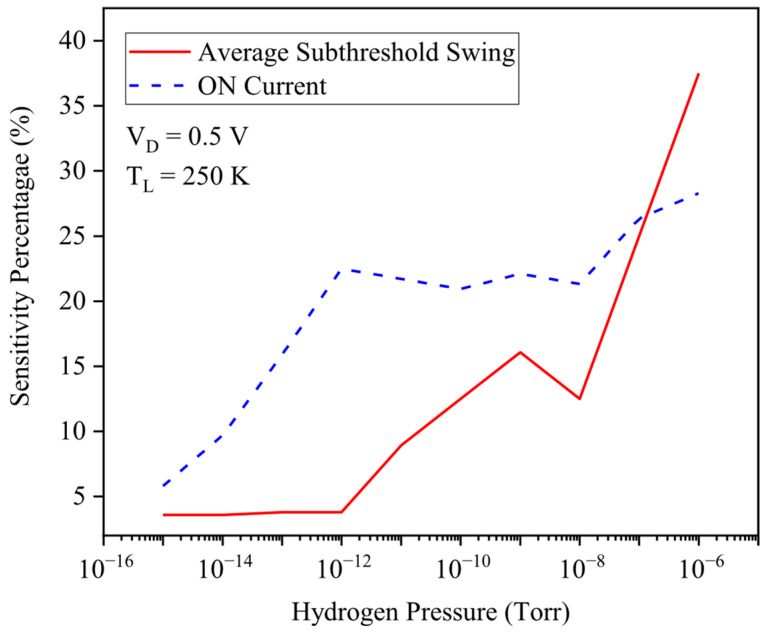
Sensitivity percentage versus hydrogen pressure. The average subthreshold swing and drain current have been utilized.

Not only has the threshold voltage criterion been focused on, but Figure 9 also evaluates hydrogen sensing using two other metrics: drain current sensitivity and average subthreshold swing sensitivity percentage, both plotted versus H_2_ pressure. At ultra-low pressures ($10^{-15}$–$10^{-8}$ Torr), drain current sensitivity dominates because small hydrogen-induced barrier reductions cause exponential increases in carrier transmission through the channel. As pressure increases further, the gate barrier is strongly lowered, the OFF current at $V_{G}=0$ rises, and the transfer curve is reshaped, giving a sharp increase in subthreshold swing sensitivity at higher H_2_ levels.

**Figure 10:**
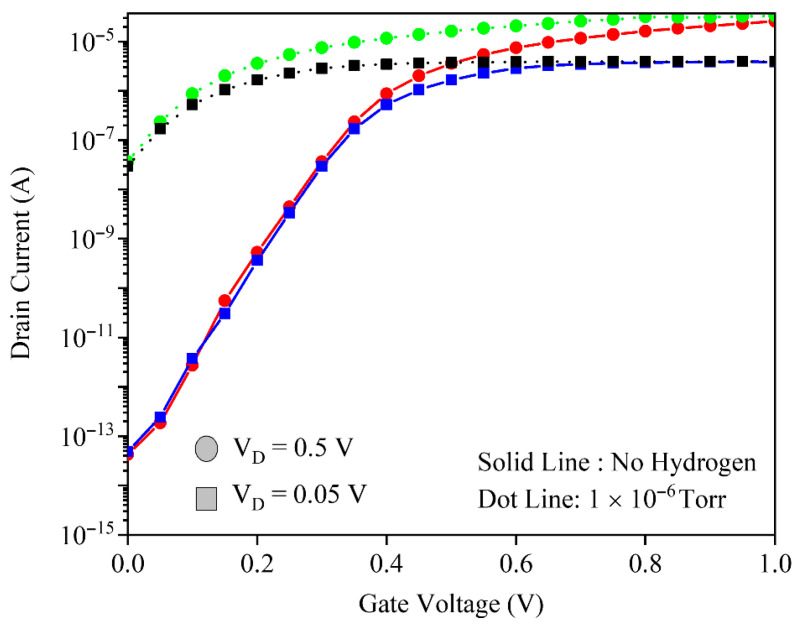
The drain current and gate voltage at hydrogen pressure of 1 × 10^−6^ Torr. Two drain voltages of 0.5 V and 0.05 V have been assumed.

The ability of the gas sensor to operate in the low-voltage regime is very important because it leads to reduced power dissipation. Figure 10 compares the transfer characteristics of the GNRFET before and after exposure to $1\times 10^{-6}$ Torr H_2_ at drain biases of 0.05 V and 0.5 V. At both biases, hydrogen induces a pronounced leftward shift mainly in the subthreshold region, while the above-threshold current changes only slightly, confirming that subthreshold transport is the primary sensing window. The similar threshold voltage and subthreshold swing sensitivities at 0.05 V and 0.5 V demonstrate that the sensor can operate effectively at low drain bias, enabling low-power hydrogen detection without sacrificing sensitivity.

**Figure 11:**
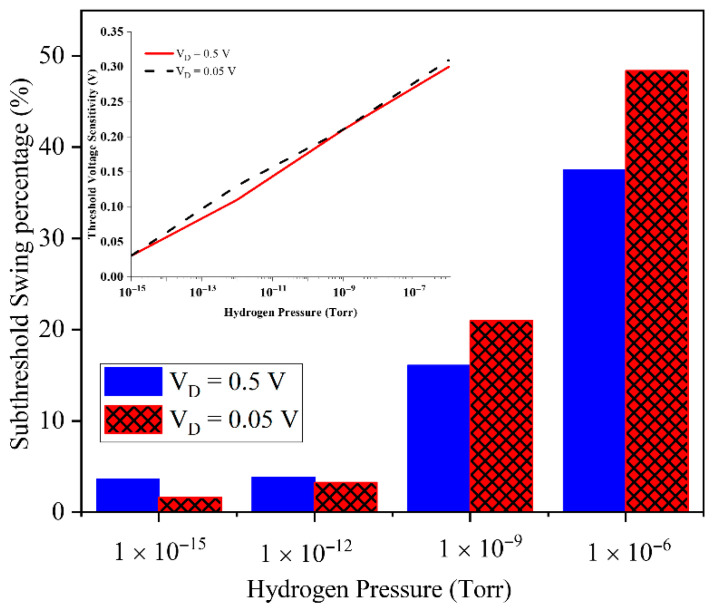
Subthreshold swing percentage versus hydrogen pressure for drain voltages of 0.05 V and 0.5 V. The threshold voltage sensitivity has been shown in the inset of the figure.

To further investigate the role of the drain bias in sensitivity, Figure 11 compares the average subthreshold swing sensitivity percentage versus the hydrogen pressure for $V_{D}=0.05$ V and 0.5 V, with the inset showing threshold voltage sensitivity. The inset indicates that $V_{TH}$ sensitivity is almost identical for both drain biases, confirming a mainly gate-controlled mechanism. In contrast, the average subthreshold swing sensitivity is higher at 0.05 V because H_2_ more strongly perturbs deep-subthreshold conduction, where small barrier changes give large relative current variations. However, this metric relies on ultralow OFF currents that approach noise and measurement limits, so $V_{D}=0.5$ V provides a more robust compromise between high sensitivity and an acceptable signal-to-noise ratio.

**Figure 12:**
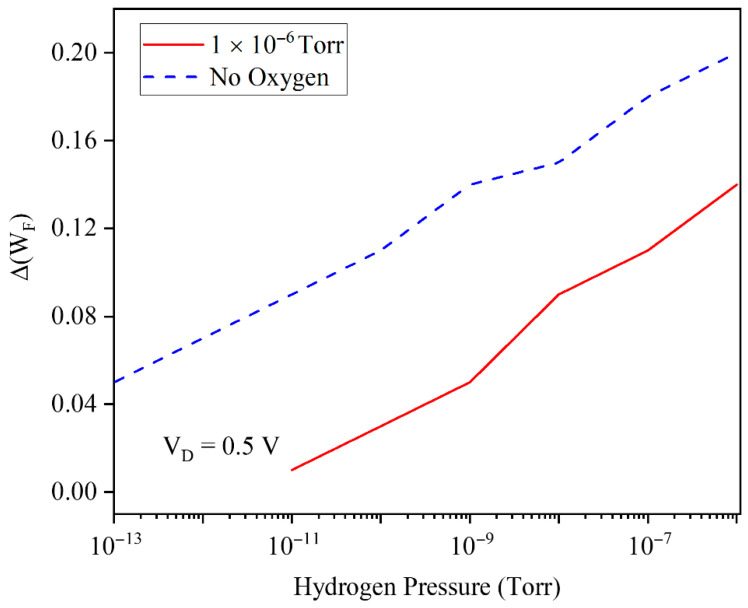
Gate work-function shift as a function of H_2_ pressure. Conditions with and without oxygen have been assumed.

The effect of background oxygen gas on sensing performance is very critical and must be investigated. Figure 12 examines the impact of background oxygen on the Pd gate work-function shift as a function of H_2_ pressure from $10^{-13}$ to $10^{-6}$ Torr. In the absence of O_2_, $\Delta W_{F}$ increases more strongly with H_2_ pressure, while in the presence of $1\times 10^{-6}$ Torr O_2_, the entire curve is shifted to lower $\Delta W_{F}$ values, indicating competitive adsorption on Pd sites and partial screening of the H-induced dipole. Consequently, the effective work-function modulation and thus the sensor sensitivity are reduced under oxygen-rich ambient conditions, which is critical for reliability assessment in realistic environments.

**Figure 13:**
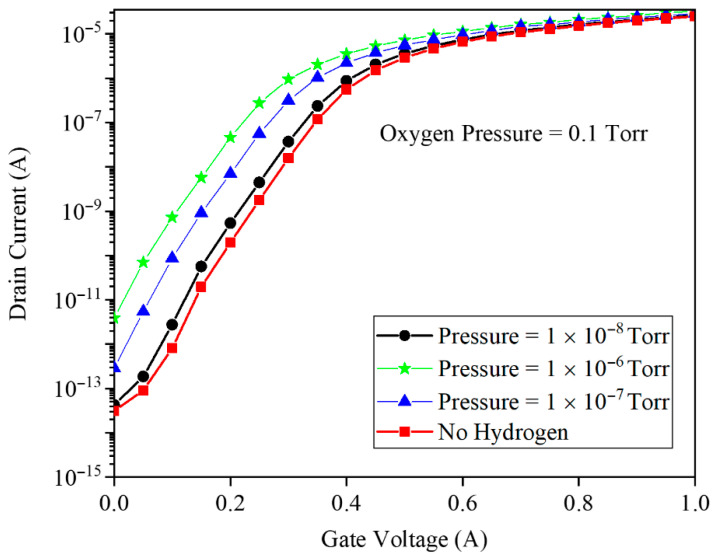
The drain current and the gate voltage for different hydrogen pressures. 0.1 Torr oxygen pressure has been assumed.

Changes in the gate work function caused by background oxygen directly affect channel conduction in the gas sensor. Figure 13 shows the transfer characteristics of the GNRFET in an O_2_ background of 0.1 Torr for H_2_ pressures of $10^{-8}$, $10^{-7}$, and $10^{-6}$ Torr. The main modulation appears in the subthreshold region, where the drain current shifts leftward with increasing H_2_, while the above-threshold current remains almost unchanged. This confirms that, even under high O_2_ ambient, the device preserves clear H_2_ sensitivity, with higher H_2_ pressures producing more pronounced subthreshold current shifts and thus a higher and more effective sensing response.

**Figure 14:**
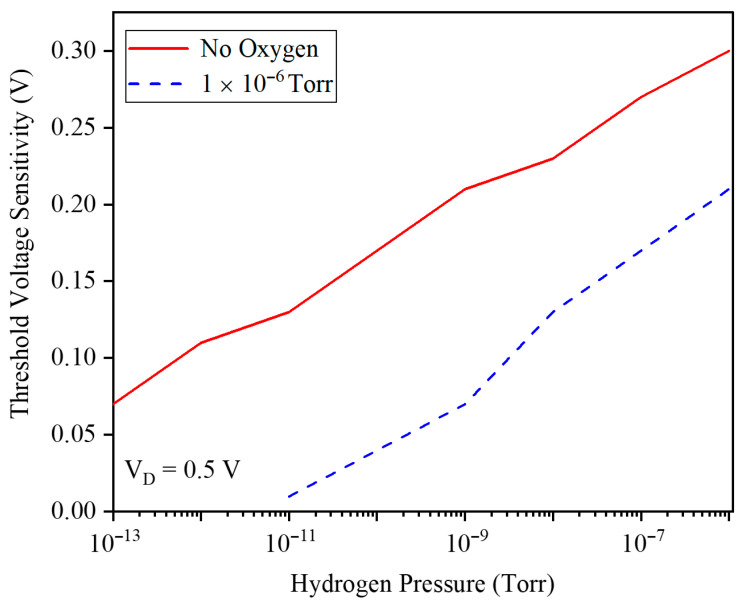
Threshold voltage sensitivity and hydrogen pressure for cases with and without background O_2_.

To further illustrate the role of oxygen in modulating sensitivity, Figure 14 plots the threshold voltage sensitivity versus hydrogen pressure for cases with and without background O_2_. In the absence of O_2_, the sensitivity remains higher over the entire pressure range, whereas introducing O_2_ reduces *V_TH_* sensitivity, with the largest deviation appearing at low H_2_ pressures. Thus, competitive O_2_ adsorption degrades the GNRFET sensor’s response in the ultratrace regime.

**Figure 15:**
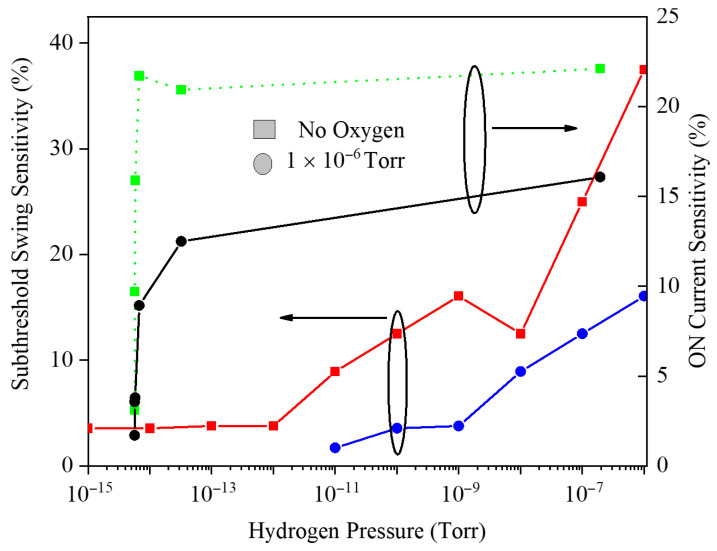
Subthreshold swing sensitivity and drain current sensitivity versus hydrogen pressure for cases with and without O_2_.

Figure 15 compares the drain current sensitivity and the average subthreshold swing sensitivity percentage versus H_2_ pressure from $10^{-15}$ to $10^{-6}$ Torr for cases with and without $10^{-6}$ Torr background O_2_. The drain current sensitivity rises sharply at ultralow H_2_ levels and then saturates, remaining systematically higher without O_2_ because competitive oxygen adsorption reduces effective work-function modulation. In contrast, the average subthreshold swing sensitivity is much less and is further degraded in the presence of O_2_; it stays nearly flat at low H_2_ and does not clearly saturate, reflecting that subthreshold conduction is primarily governed by gate work-function modulation rather than barrier lowering, making this metric less practical for trace-level H_2_ detection.

**Figure 16:**
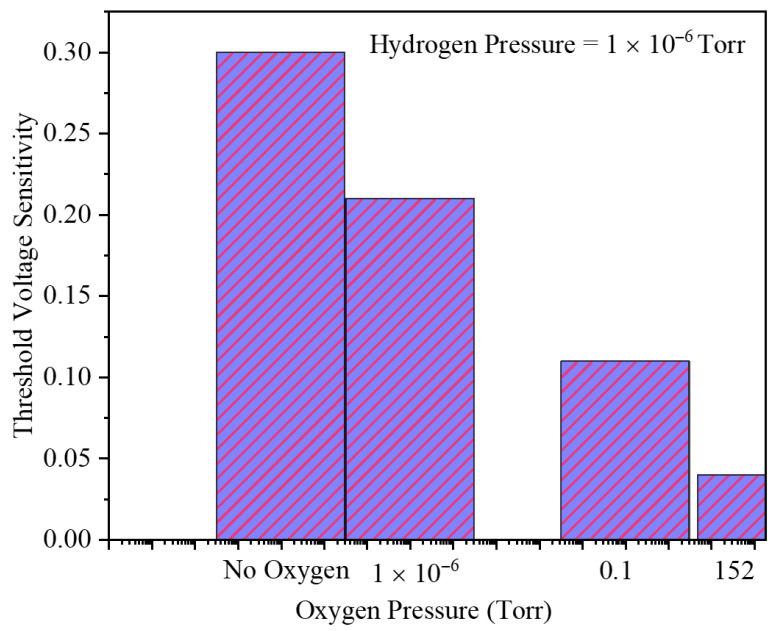
Threshold voltage sensitivity and the different O_2_ pressures at the fixed hydrogen pressure of 1 × 10^−6^ Torr.

Figure 16 depicts the threshold voltage sensitivity of the GNRFET sensor versus O_2_ pressure at a fixed H_2_ pressure of $1\times 10^{-6}$ Torr (cases: no O_2_, $1\times 10^{-6}$, 0.1, and 152 Torr). The sensitivity progressively decreases as O_2_ pressure increases, evidencing competitive adsorption on the Pd gate that partially screens the H_2_-induced work-function shift. Nevertheless, a distinct $V_{TH}$ shift persists even at 0.1 and 152 Torr O_2_, demonstrating that the GNRFET retains appreciable H_2_ sensing capability under highly oxygen-rich conditions, highlighting its robustness for realistic ambient operation.

**Figure 17:**
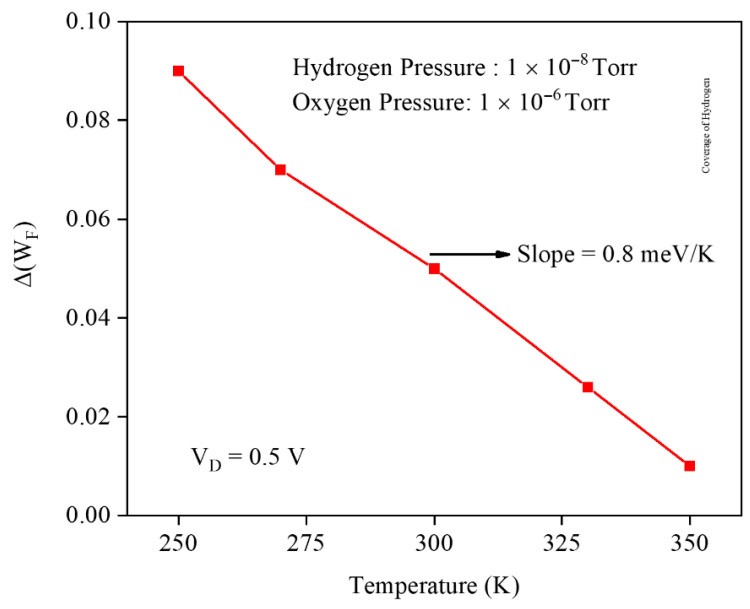
Gate work-function shift and temperature at P_H_2__ of 1 × 10^−8^ Torr and P_O_2__ 1 × 10^−6^ Torr.

Temperature critically governs gas sensor performance because adsorption–desorption kinetics, reaction rates, and carrier transport are all strongly temperature dependent. Lower temperatures can enhance adsorption and interface coverage, increasing work-function modulation but also slowing response and recovery. Higher temperatures usually accelerate desorption and reduce coverage, degrading sensitivity yet improving reversibility and stability. Therefore, optimizing operating temperature is essential to balance sensitivity, speed, selectivity, and long-term reliability in practical gas sensing applications. Figure 17 shows the Pd gate work-function variation versus lattice temperature from 250 to 350 K at $P_{H_{2}}=10^{-8}$ Torr and $P_{O_{2}}=10^{-6}$ Torr. The work-function shift decreases almost linearly with temperature, with a slope of about 0.8 meV/K, indicating that higher lattice temperature weakens hydrogen-induced dipoles and is therefore expected to degrade the GNRFET gas sensor’s sensitivity.

The inclusion of sub-room-temperature conditions (e.g., 250 K) enables a clearer separation of thermal effects from intrinsic sensing behavior. At lower temperatures, increased hydrogen coverage and reduced thermal broadening enhance subthreshold features and improve sensitivity, particularly in threshold voltage. These observations provide insight into the fundamental sensing limits. In practical devices, such operating conditions can be approximated or compensated using localized thermal control techniques, including integrated micro-heating or cooling elements.

**Figure 18:**
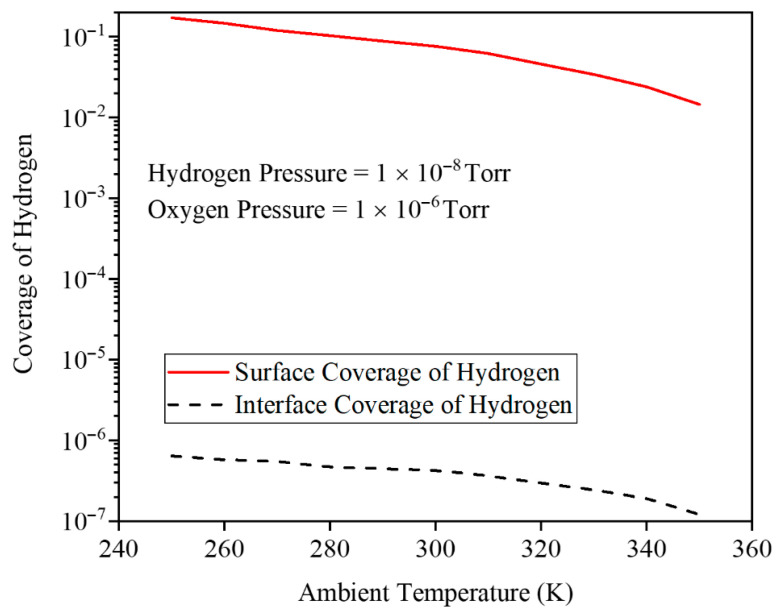
The interface/surface coverage of hydrogen versus the temperature.

To better understand the main reason for the degradation of the sensitivity of the gas sensor by temperature, Figure 18 illustrates the hydrogen surface and interface coverage decreasing with the lattice temperature at fixed H_2_ and O_2_ pressures. Because higher temperatures promote desorption and lower the sticking probability of hydrogen atoms on the catalytic lattice. As fewer H atoms remain at surface and interface sites, the number of interfacial dipoles drops, which reduces the work-function shift and thus weakens the electrostatic modulation and sensing response of the GNRFET gas sensor at elevated temperatures.

**Figure 19:**
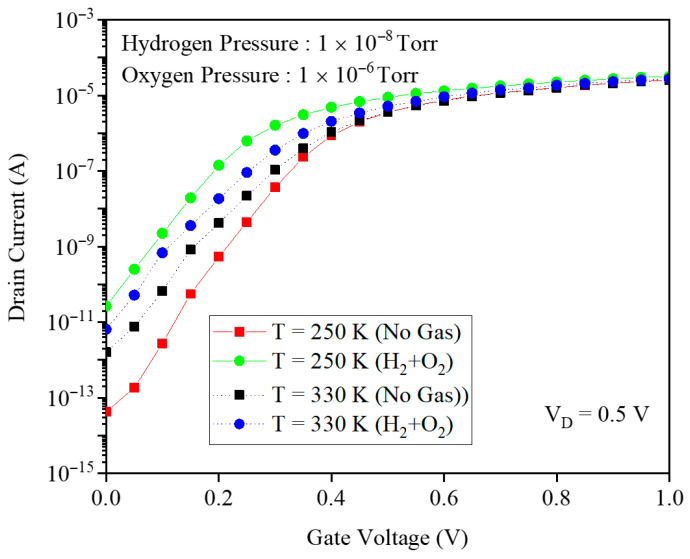
The drain current and gate voltage for temperatures of 250 and 330 K.

**Figure 20:**
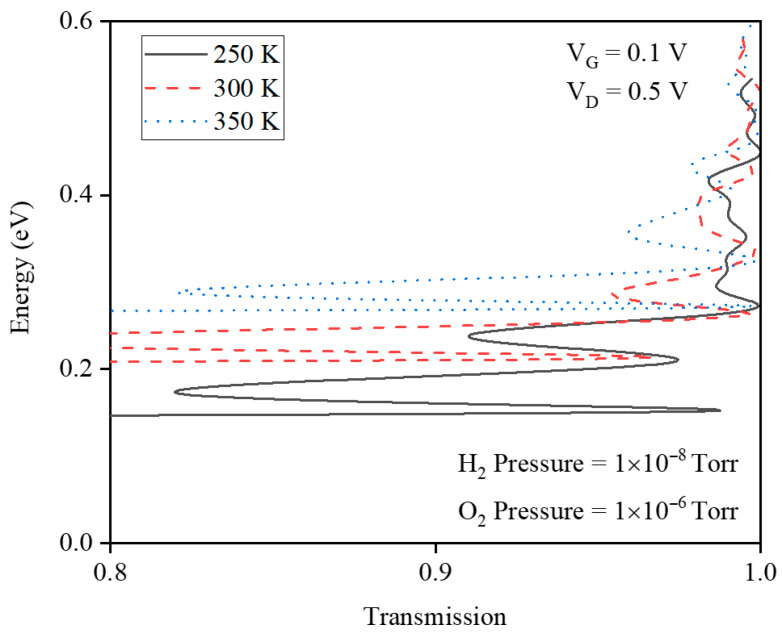
The energy-transmission profile for fixed O_2_ and H_2_ pressures at different temperatures.

Figure 19 shows the transfer characteristics of the gas sensor at $P_{H_{2}}=10^{-8}$ Torr and $P_{O_{2}}=10^{-6}$ Torr, comparing 250 K and 330 K before and after H_2_ exposure. The drain current modulation is concentrated in the subthreshold region and is stronger at the lower temperature. Without gas, an increasing temperature narrows the bandgap, enhances tunneling, and raises $I_{D}$. Under H_2_ exposure, higher temperature lowers hydrogen interface coverage, increases the Pd gate work function and $V_{TH}$, and reduces $I_{D}$, with this work-function effect dominating over the bandgap-driven tunneling increase. Figure 20 presents the transmission coefficient as a function of energy for three lattice temperatures, 250, 300, and 350 K, at fixed H_2_ and O_2_ pressures. At 250 K, the hydrogen interface coverage on the Pd gate is the highest, so the Pd–H dipoles strongly lower and smooth the source–channel potential barrier. This pulls several resonant transmission peaks down toward the Fermi level, expanding the low-energy window where transmission is high and thus significantly enhancing the drain current. As the temperature increases to 300 and 350 K, hydrogen desorbs from the interface, reducing coverage and weakening the dipole field. The effective gate work function increases, the barrier reforms, and many low-energy resonant states shift to higher energies, so the low-energy transmission baseline and the number of accessible peaks both decrease. Consequently, the integrated transmission over the thermally occupied energy range shrinks, which directly explains the reduced drain current and degraded sensing response at elevated temperatures.

**Figure 21:**
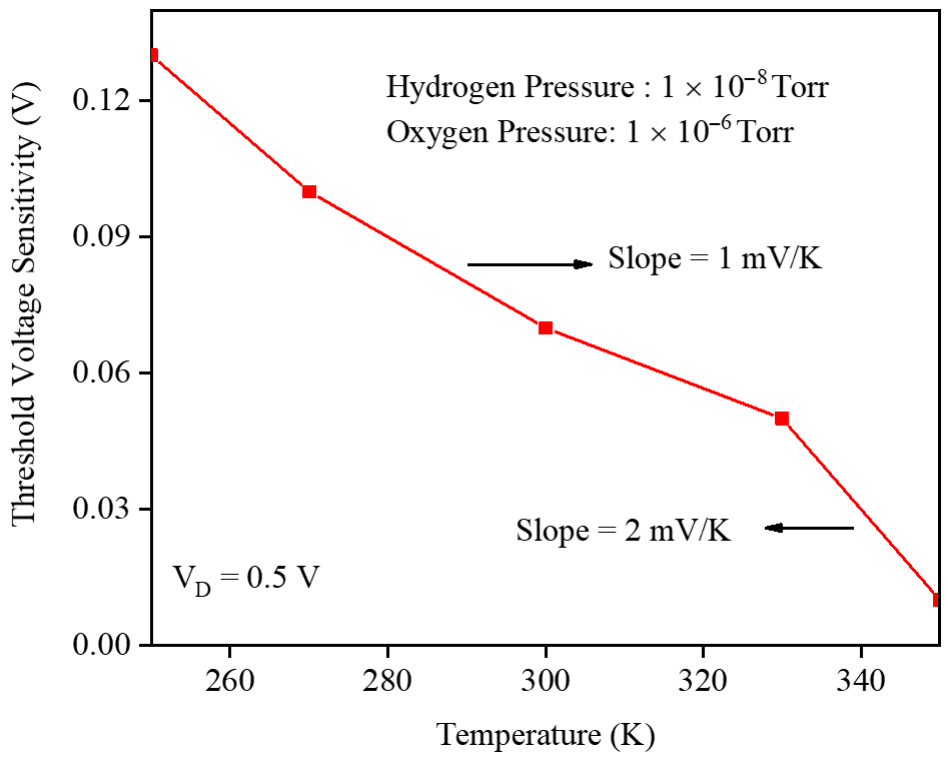
Threshold voltage sensitivity and the temperature at fixed H_2_ and O_2_ pressures.

To quantify the temperature effect on the gas sensor, Figure 21 shows the threshold voltage sensitivity as a function of temperature at $P_{H_{2}}=10^{-8}$ Torr and $P_{O_{2}}=10^{-6}$ Torr. The sensitivity decreases with increasing temperature, with a gentler slope of about 1 mV/K at lower temperatures that steepens to roughly 2 mV/K at higher temperatures, indicating that an elevated lattice temperature strongly suppresses the H_2_-induced $V_{TH}$ shift and can seriously degrade the overall sensing performance of the GNRFET hydrogen gas sensor.

The gate length and GNR width, which change by varying the GNR index, have a key impact on gas sensitivity, because they control electrostatics, bandgap, and transport regime. For this reason, the subsequent paragraphs are related to the sensitivity that is dependent on these main parameters.

**Figure 22:**
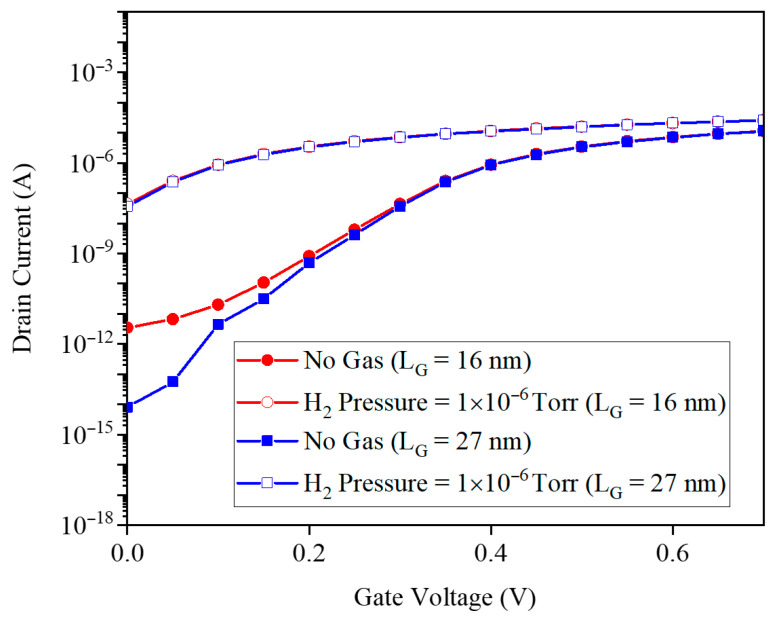
The drain current and gate voltage for gate lengths of 16 and 27 nm.

In continuation, the aim is to measure the sensitivity dependence on geometry changes in the gas sensor. Two important parameters related to geometry in terms of gate length and GNR width have been considered in this investigation. Figure 22 presents $I_{D}$–$V_{G}$ characteristics for gate lengths $L_{G}=12$ nm and 27 nm at $P_{H_{2}}=10^{-6}$ Torr, before and after H_2_ exposure. In the ON region, currents are nearly identical for both lengths and both gas conditions, indicating that ballistic quantum transport and ribbon width, rather than $L_{G}$, set the ON current. In contrast, subthreshold slopes differ: the longer gate (27 nm) shows a gentler pre-exposure slope, making subthreshold swing-based sensitivity the key length-dependent metric, while post-exposure currents converge because work-function modulation dominates over gate-length effects.

**Figure 23:**
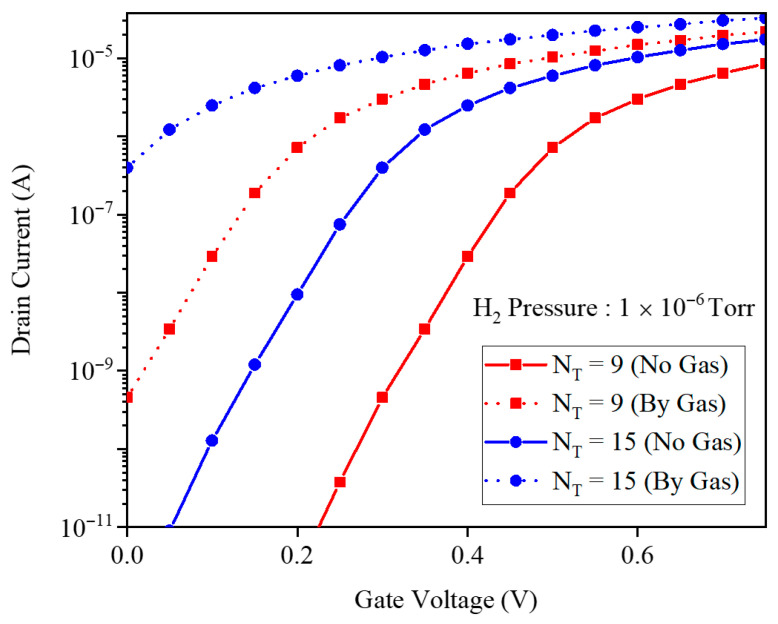
Drain current and gate voltage for different GNR widths by indexes of 9 and 15.

Figure 23 shows the drain current versus gate voltage before and after exposure to $1\times 10^{-6}$ Torr H_2_ for two GNR widths with indexes 9 and 15. Both above-threshold and subthreshold regions depend on nanoribbon width, because changing the index modifies the bandgap and thus the absolute drain current and sensor sensitivity. The most pronounced width-induced variation appears in the subthreshold swing, indicating that subthreshold-regime sensitivity is particularly sensitive to nanoribbon index engineering.

**Figure 24:**
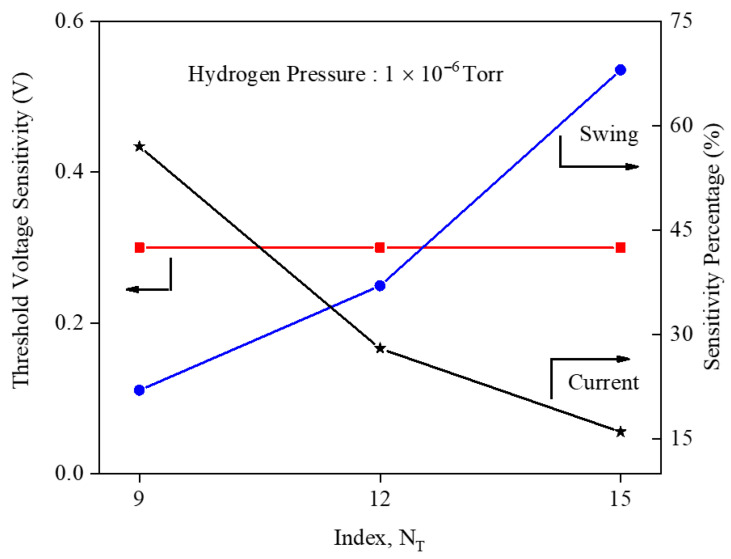
Sensitivity versus different GNR indexes.

The threshold voltage sensitivity and sensitivity percentage for the different indexes have been illustrated in Figure 24. The figure emphasizes that each sensitivity metric responds differently to nanoribbon width engineering, so the “best” GNR index depends on the chosen figure of merit. Drain current sensitivity decreases with an increasing index because wider ribbons carry larger baseline current, reducing the relative $\Delta I_{D}/I_{D}$. The subthreshold swing sensitivity increases with the index since a higher subthreshold current lowers the swing and amplifies its fractional change. Threshold voltage sensitivity remains nearly constant across indexes, making $V_{TH}$ shift the most robust and fabrication-tolerant metric for practical GNRFET hydrogen sensors.

**Figure 25:**
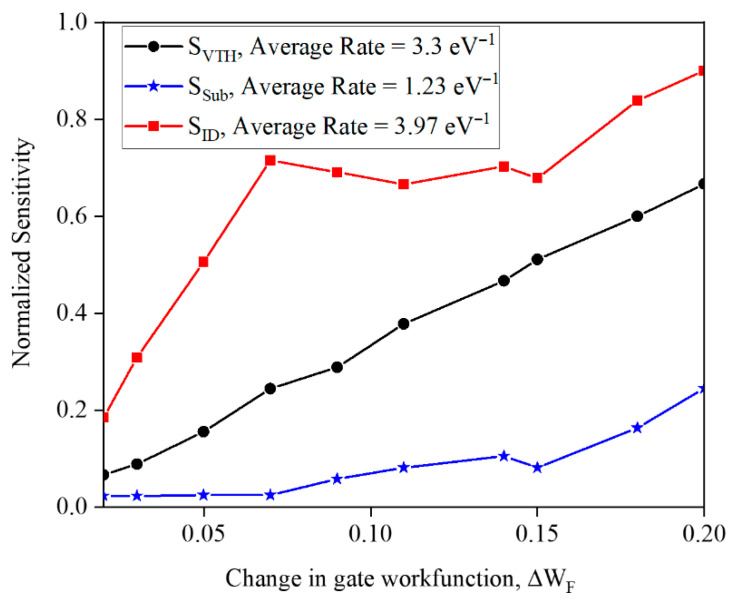
Normalized sensitivity versus Δ*W_F_*. The variation rate is seen in the inset of the figure.

As we know, the effect of the types of background different gases on the sensor is modeled by a change in the gate Fermi level giving $\Delta W_{F}$ according to the equation below [26,43]:

(11) $$\Delta W_{F}=\left(K_{B}T/2\beta \right)Ln\left(P/\sum_{i}k_{i}P_{i}+1\right)$$

where $\beta =\gamma \left(E_{F}-\chi_{g}\right)$, *E_F_* is the Fermi level of the gate, $\chi_{g}$ is the Mulliken electron-negativity coefficient of the target gas, *P_i_* is the concentration of background gases, and *k_i_* is the selectivity coefficient. It is worth noting that an increase in the different background gases has led to a decrease in $\Delta W_{F}$. In order to show this effect, a different range of $\Delta W_{F}$ is assumed, and the average variation rate of sensitivity (average slope) can be introduced as a metric to quantify the power of selectivity. It is pointed out that a low average slope of the curve is desirable. Figure 25 illustrates the normalized sensitivity upon $\Delta W_{F}$ based on the sensitivity that is based on threshold voltage, subthreshold swing, and drain current. The lowest variation rate is for *S_Sub_* with a value of 1.23 eV^−1^, and the highest value is for *S_ID_*. It can be implicitly understood from this finding that the different background gas effects on *S_Sub_* are lower compared to those on *S_VTH_* and *S_ID_*.

Having a low response time, *t_r_*, is very essential in sensing gas molecules. A theoretical relation to quantify *t_r_* is defined as the following equation [40]:

(12) $$t_{r}=K\frac{SS_{avg}}{\rho }$$

where parameter *K* is a process-related constant, *SS_avg_* and $\rho_{0}$ are the average subthreshold swing and gas molecule concentration, respectively. It is worth noting that since the gas sensor under investigation is based on the GNRFET device, which is renowned as a very low subthreshold device, *t_r_* is expected to be reduced according to Equation (12).

## 5. Statistical Analysis

In order to investigate the reliability of the proposed hydrogen gas sensor, a rigorous statistical analysis has been performed on the normalized sensitivities based on threshold voltage (*S_VTH_*), subthreshold swing (*S_Sub_*), and drain current (*S_ID_*), as illustrated in Table 1. For this reason, the different physical parameters in terms of gate length (*L_g_*), gate oxide thickness (*t_ox_*), ambient temperature (*T_L_*), drain voltage (*V_d_*), and drain/source doping density (*N*_*S*/*D*_) have been changed to generate numerous samples of the gas sensors. The variation ranges of these parameters have been given in the table. For each parameter, the mean (*µ*) and standard deviation (*δ*) have been extracted as shown in the table. In order to evaluate the sensitivity scattering rate, the variation percentage (*VP*) coefficient is defined as follows:

(13) $$VP=\frac{\delta }{\mu }*100$$

The table gives interesting information about the reliability and uncertainty of the gas sensor. For all the different samples, the CV coefficient based on *S_VTH_* is the lowest value, and the highest value is based on *S_ID_*. This indicates that physical parameters have a low effect on the sensitivity based on threshold voltage. Hence, *S_VTH_* is introduced as a powerful criterion for the evaluation of the sensitivity of the gas sensor. It is worth noting that the temperature effect is still a key factor to deviate the CV even based on *S_VTH_* to high values since the interface and surface coverage of hydrogen is seriously affected by the temperature. According to Table 1, the CV based on *S_ID_* experiences a high value, showing that sensitivity related to drain current has considerable dependence on the physical parameters of the gas sensor, in spite of its high absolute value. Hence, for reaching a more reliable gas sensor, *S_ID_* should not be used.

Regarding *S_VTH_*, it is evident from the table that the gas sensor has given the highest immunity to the different drain voltages and gate oxide thickness (less than 2%). Hence, it is possible to reduce the drain voltage for the aims of low power and to reduce the gate oxide thickness for the aim of ultrascaling without cost on the sensing power. Regarding *S_Sub_*, the gas sensor immunity on gate oxide thickness, drain/source doping density, and drain voltage is high, enabling us to change these parameters.

## 6. Comparative Analysis of Findings

Performing a comparative analysis of simulation, four important categories, including subthreshold-dominated transport, metric hierarchy for sensing, temperature as the dominant environmental factor, and geometric optimization, have been considered to give more useful information.

*Subthreshold-Dominated Transport as Core Mechanism:* The initial pressure-dependent work-function and transmission studies establish that hydrogen sensing is fundamentally a subthreshold phenomenon (sensitivity based on threshold voltage and subthreshold swing) driven by a Pd–H work-function modulation that reshapes the source–channel potential landscape. The dramatic transmission peak reorganization and local density of states enhancement at low energies occur precisely because the hydrogen-lowered barrier pulls quasi-bound states down to the Fermi window. This insight implies that any sensor architecture abandoning subthreshold operation—operating deeply into saturation or at elevated gate bias—surrenders the core sensitivity mechanism, regardless of other optimizations.

*The Metric Hierarchy for Practical Sensing:* The drain current sensitivity, evaluated near and slightly above the threshold, increases with hydrogen pressure but tends to saturate and depends strongly on the exact bias point, so its numerical value is less robust for comparison across devices and operating conditions. Subthreshold swing sensitivity can, in principle, be enhanced by geometric tuning, yet in practice it requires precise extraction from small-current data and is more susceptible to measurement uncertainty. Average subthreshold swing sensitivity is theoretically maximizable through geometric tuning but operationally impractical because it requires measuring tiny current variations in a regime dominated by noise. In contrast, the threshold voltage sensitivity reaches about 300 mV at $10^{-6}$ Torr H_2_ without O_2_, shows an almost linear dependence on pressure, and remains nearly invariant with the gate length and nanoribbon index; this geometric immunity makes the threshold-shift metric the most reliable choice for practical, array-deployable GNRFET hydrogen sensors.

*Temperature as Dominant Environment Factor:* Temperature-dependent measurements reveal that temperature is a more severe degradation source than background O_2_. While 152 Torr O_2_ reduces ΔVTH from 300 mV to 40 mV (a 7× penalty), temperature elevation from 250 to 350 K causes both interface coverage collapse and low-energy transmission window shrinkage, producing steeper degradation curves. This suggests practical sensors should prioritize cryogenic or active cooling over O_2_ scrubbing if extreme sensitivity is required. Moreover, the removal of background oxygen can enhance the sensitivity, resulting in an ultra-trace detection level.

*Uncertainty analysis:* A rigorous statistical analysis assessed the reliability of a hydrogen gas sensor by examining normalized sensitivities based on threshold voltage (*SV_TH_*), subthreshold swing (*S_Sub_*), and drain current (*S_ID_*). Physical parameters—including gate length, oxide thickness, temperature, drain voltage, and doping density—were varied to generate multiple samples. The variation percentage (VP) coefficient revealed that SV_TH_ exhibited the lowest scattering, indicating minimal dependency on physical parameters and superior reliability. In contrast, S_ID_ showed high variability, disqualifying it for robust sensing. Temperature remained a critical factor affecting even *SV_TH_*. *SV_TH_* is thus recommended as the most dependable sensitivity metric.

## 7. Conclusions

This paper demonstrates that Pd-gated GNRFETs can achieve strong hydrogen sensitivity through interface coverage-driven work-function modulation coupled with quantum-confined transport. At $10^{-6}$ Torr H_2_, the maximum threshold voltage shift reaches about 300 mV in oxygen-free ambient but drops to around 40 mV at 152 Torr O_2_, showing that competitive adsorption substantially reduces, but does not eliminate, the sensing response and indicating stable sensing behavior within the limited environment conditions studied. The main current modulation resides in the subthreshold regime, where barrier lowering and resonant transmission produce large relative drain current changes. Structural tuning via gate length and GNR width can selectively change subthreshold swing and current-based sensitivities, while the threshold voltage sensitivity remains nearly constant across geometries, making it the most reliable metric for device-to-device comparison and calibration. Temperature primarily acts by decreasing hydrogen coverage and shrinking the low-energy transmission window, thereby degrading all sensitivity metrics at elevated values. The investigation of the dependence of sensitivity on background oxygen gas pressure and ambient temperature has demonstrated that the role of temperature must receive greater attention than the influence of background oxygen in considerations related to gas sensor sensitivity. The statistical analysis demonstrated that the physical parameters have the least effect on *S_VTH_*. Overall, the results provide quantitative benchmarks and clear design guidelines for optimizing geometry, biasing, and operating ambient of GNRFET-based hydrogen sensors for low-power, reliable detection in realistic oxygen-containing environments.

## Figures and Tables

**Table 1:** Statistical analysis results of the gas sensor. The *VP* coefficient is measured to quantify reliability.

Parameters	Ranges	Mean, *µ*		Standard Deviation, *δ*		Variation Percentage
*L_g_*	[9–34] nm	*S_VTH_*	0.96	*S_VTH_*	0.02	2.1%
		*S_Sub_*	0.58	*S_Sub_*	0.31	52%
		*S_ID_*	0.48	*S_ID_*	0.45	94%
*t_ox_*	[1–3] nm	*S_VTH_*	0.96	*S_VTH_*	0.01	1.66%
		*S_Sub_*	0.79	*S_Sub_*	0.15	19.5%
		*S_ID_*	0.49	*S_ID_*	0.27	56.1%
*T_L_*	[250–350] K	*S_VTH_*	0.55	*S_VTH_*	0.29	53.5%
		*S_Sub_*	0.4	*S_Sub_*	0.31	77.4%
		*S_ID_*	0.46	*S_ID_*	0.32	70.5%
*V_d_*	[0.05–0.8] V	*S_VTH_*	0.96	*S_VTH_*	0.01	1.9%
		*S_Sub_*	0.68	*S_Sub_*	0.14	20.4%
		*S_ID_*	0.46	*S_ID_*	0.4	86.8%
*N*_*S*/*D*_	[1.8–6] × 10^8^ /m	*S_VTH_*	0.97	*S_VTH_*	0.02	2.13%
		*S_Sub_*	0.73	*S_Sub_*	0.16	22.6%
		*S_ID_*	0.68	*S_ID_*	0.33	48.2%

## Data Availability

The original contributions presented in this study are included in the article. Further inquiries can be directed to the corresponding authors.
